# [Retracted] Effects and mechanisms of blocking the hedgehog signaling pathway in human gastric cancer cells

**DOI:** 10.3892/ol.2026.15844

**Published:** 2026-09-03

**Authors:** Hongbing Gu, Xu Li, Congzhi Zhou, Yugang Wen, Yang Shen, Lisheng Zhou, Jikun Li

Oncol Lett 9: 1997–2002, 2015; DOI: 10.3892/ol.2015.3032

Following the publication of the above paper, it was drawn to the Editor's attention by a concerned reader that, regarding the cell invasion assay experiments shown in Fig. 2A on p. 2000, the ‘10 μM cyclopamine’ data panel was strikingly similar to data that were included in a paper written by different authors at different research institutes that had already been accepted for publication in the journal *Oncology Letters*.

Owing to the fact that the contentious data mentioned above had already been accepted for publication before this paper was received at *Oncology Letters*, the Editor has decided that it should be retracted from the Journal. The authors were asked for an explanation to account for these concerns, but the Editorial Office did not receive a reply. The Editor apologizes to the readership for any inconvenience caused.

